# CORESS Feedback: Cases from the Confidential Reporting System for Surgery

**DOI:** 10.1308/rcsann.2026.0092

**Published:** 2026-09-01

**Authors:** HJ Corbett, W Hawkins

**Affiliations:** on behalf of the CORESS Advisory Board

## Abstract

CORESS is an independent charity. Past CORESS cases can now be identified using a searchable CORESS case database, available via our website (coress.org.uk). We are grateful to those who have provided the material for these vignettes, some of which come from NHS England and Getting It Right First Time reports. The online reporting form is available on the CORESS website, which also includes previous Feedback reports. Published cases are acknowledged by a Certificate of Contribution, which may be included in the contributor’s record of continuing professional development, or which may form part of appraisal or annual review of competence progression portfolio documentation. Contributions from surgeons in training are particularly welcome.

## Pitfalls of automation

### Case 334

A 68-year-old man underwent a radical prostatectomy to treat prostate cancer. As part of his postoperative surveillance, the patient’s prostate-specific antigen (PSA) was measured. The result was “within the normal range” and the hospital’s automated results system acknowledged the result on behalf of the clinical team. The PSA reading was actually high for a patient who had undergone a prostatectomy and was a sign of residual disease. Thankfully, the abnormal result was picked up during an audit of the cancer care pathway; the patient might have had a significant delay in further treatment had that not happened.

### Reporter’s comments

The automatic result acknowledgement process did not have the right rules in place for this scenario. A manual check process has since been introduced.

### CORESS comments

It is established that if you order a test, you are responsible for checking the result. All test results must be reviewed in context. It is notable that even if this result had been checked in person, the context could have been missed. However, automated systems can and should be programmed to allow for scenarios such as this. Further consideration should be given to the term “normal range” as test results are often dependent on many factors, including age, sex and current or past medical treatment. Use of “reference range” may be more appropriate and attention to the pathologist’s comments is essential.

## Unexpected retained swab after knee replacement

### Case 335

A 65-year-old woman underwent total knee replacement. The procedure was straightforward and the swab counts were recorded as correct at the end of the procedure. The following day, the routine postoperative x-ray revealed that there was a radio-opaque foreign body in the knee joint. The patient returned to the operating theatre for removal of the foreign body, which imaging revealed to be in the suprapatellar pouch. Arthroscopy was performed and the foreign body was found to be the radio-opaque thread from a swab.

### CORESS comments

Retention of a foreign body is a ‘never event’ and while the case details reported to CORESS were brief, an investigation no doubt resulted. The CORESS advisory board considered factors that may have led to the separation of a thread (or threads) from a swab, such as the quality of the swabs and the possibility that the swab had been cut to reduce it in size. The lesson here is the importance of checking the integrity of any item in the surgical field to minimise the risk of inadvertently leaving part of an item behind, especially for items that have been cut or become frayed. When items are cut or trimmed, this should be acknowledged by the whole scrub team and documented on the theatre whiteboard.

## Medication error: inadvertently high concentration of heparin

### Case 336

A patient who had undergone a femoral popliteal bypass using reversed great saphenous vein the previous week was admitted with pain and swelling in the groin. Computed tomography angiography identified bleeding from the proximal anastomosis. The patient was taken to the operating theatre by two experienced consultant vascular surgeons and a vascular registrar. The scrub nurse for the case was also experienced and regularly scrubbed for vascular cases. The case proceeded according to the preoperative plan: ipsilateral external iliac control was achieved and the bypass was found to be bleeding from the anastomosis, where a stitch had likely torn through. The bypass was fully disconnected, the common femoral artery, the profunda femoris artery and the superficial femoral artery were controlled, and 3,000IU of unfractionated heparin were given.

In order to prevent ‘clot-off’ while the bypass was disconnected, a 4Fr arterial catheter was sited into the bypass for administration of an infusion of heparinised saline from within the operative field. The operation proceeded without incident although during closure, it was noted that the patient was bleeding excessively from all ‘raw’ surfaces. Thromboelastography was performed and the patient’s haemoglobin concentration was checked. The patient required 2 units of packed red cells, 2 units of cryoprecipitate and 2 units of fresh frozen plasma to maintain cardiovascular stability and correct the clotting defect.

While completing the operation note, the registrar asked the scrub nurse to confirm the volume and concentration of heparinised saline used in the bypass. At this point, it was realised that a heparin concentration of 10,000 units in 1 litre (10 units/ml) had been used.

### Reporter’s comments

Neither of the consultants nor the registrar were aware of the greater than intended dose of heparin being administered. The person who was aware (the scrub nurse) did not voice any concern or bring to the attention of the operating surgeons the large dose of heparin being administered. The ability of all team members to speak up and raise concerns is critical to maintaining patient safety. For whatever reason, in this case, the scrub nurse presumed that the two consultants and registrar were aware of the medication they were administering.

### CORESS comments

Communication between members of the theatre team must be clear and thorough. Where possible, plans for administration of intraoperative medication should be clarified at the team brief and ‘time out’ check, and the whole team should be made aware when the medication is being given. Documentation on the theatre whiteboard is advised. When unplanned, there should be a clear discussion among the team regarding the medication and dose preparation to reduce the risk of inadvertent overdose or interactions between medications. Pre-filled syringes can help reduce the cognitive burden that can increase the risk of such events.

The following case is taken with permission, and with thanks, from Learning from Surgical Mistakes (www.lfsm.co.uk).

## Bone cement implantation syndrome

### Case 337

An 85-year-old woman was brought to the operating theatre for a left hip hemiarthroplasty following a displaced intracapsular neck of femur fracture. Her admission Abbreviated Mental Test Score was 3/10 and consent was documented on a Form 4 owing to her lack of capacity. A ‘do not attempt cardiopulmonary resuscitation’ (DNACPR) form had been completed preoperatively with the patient’s next of kin.

Two experienced registrars were performing the surgery. The consultant was in the hospital but not in the operating theatre. During cementation, the patient developed ventricular tachycardia and lost her cardiac output. Management of the situation was ‘by the book’: the anaesthetist gave the order to press the emergency buzzer, the wound was quickly closed using skin clips and covered with a large Ioban^™^ dressing, the patient was turned supine and cardiopulmonary resuscitation (CPR) was commenced with brief return of cardiac output.

Ultimately, the event was unsurvivable but prompt action bought enough time for the patient’s family to attend theatre. A team debrief was conducted and the decision made to stand down the theatre.

### Reporter’s comments

Teamwork and communication worked well during this incident; the problem (bone cement implantation syndrome) was quickly recognised. It is good practice for the surgeon to warn the anaesthetist prior to cementation. The decision to carry out CPR on a patient with a DNACPR form should ideally be discussed well before the situation arises. If the surgeon and anaesthetist agree, it is reasonable to attempt CRP while seeking to correct reversible causes. In this case, it allowed time for the family to attend, which was appreciated.

### CORESS comments

A fractured neck of femur in such a patient may be considered to be a life-limiting event, and in this context, non-operative management with symptom relief would have been reasonable. The family should be counselled accordingly. If opting for surgical management, a plan should be made for management of perioperative events such as this.

## Another disappearing drain: the perils of ‘cutting and bagging’ drains

### No case number

The patient had an abdominal drain placed during a laparotomy. The drain was shortened by the doctors during the ward round. A few hours later, the nurse caring for the patient went to change the bag over the drain as it was leaking and they noted that the visible drain was quite short. The nurse went to get some pain relief and antiemetics for the patient. On their return, the patient’s husband advised that the drain bag was leaking again. The site was checked but the drain had disappeared, with only the stitch present outside. The medical staff were called; ultimately, the patient required a return to the operating theatre to retrieve the drain.

### CORESS comments

**Figure 1:**
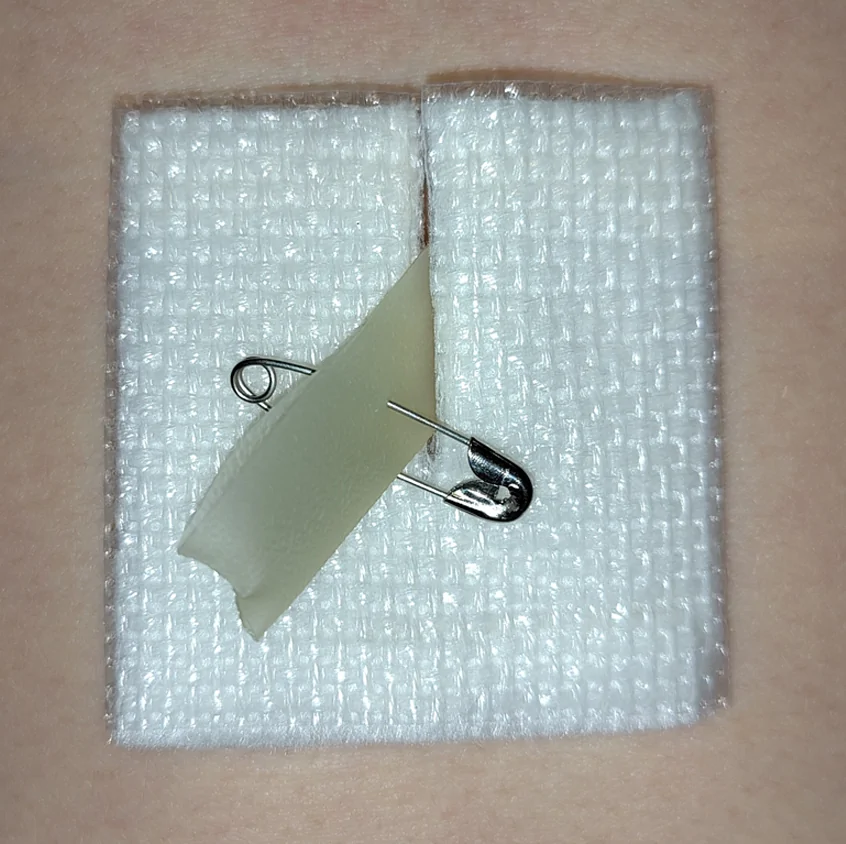
A sterile safety pin placed through the drain just above the level of the skin

This is unfortunately a recurring issue that results in the need for further surgery to retrieve the drain tubing. Retraction of drains is avoidable: a sterile safety pin should be placed through the drain just above the level of the skin (Figure 1) as this, unlike sutures, can be easily re-sited when the drain length needs to be adjusted. The Association of Surgeons of Great Britain and Ireland has produced useful guidance for wound drain shortening.^1^

Note that the critical steps in the procedure include:

- Ensuring adequate protrusion – cut the drain at least 10 cm from the patient’s skin
- Placement of a sterile safety pin through the drain as close as possible to the skin (to prevent drain retraction) but not under tension against the skin, where it could cause a pressure injury
- Clear documentation of the drain length from skin exit site to the end of the drain end, as well as the site of drainage
